# Hair Cortisol in Young Children with Autism and Their Parents: Associations with Child Mental Health, Eating Behavior and Weight Status

**DOI:** 10.1007/s10803-024-06672-0

**Published:** 2025-01-22

**Authors:** Anna van der Lubbe, Hanna Swaab, Erica van den Akker, Robert Vermeiren, Wietske A. Ester

**Affiliations:** 1 Sarr Autism Rotterdam, Youz Child- and Adolescent Psychiatry, Parnassia Group, Dynamostraat 18, Rotterdam, The Netherlands; 2 Clinical Neurodevelopmental Sciences, Leiden University, Wassenaarseweg 52, Leiden, The Netherlands; 3 Parnassia Group, Parnassia Academy, Den Haag, The Netherlands; 4 Leiden Institute for Brain and Cognition, Leiden University, Leiden, The Netherlands; 5 Department of Pediatrics, Division Pediatric Endocrinology and Obesity Center CGG NL, Erasmus University Medical Centre-Sophia Children’s Hospital, Rotterdam, The Netherlands; 6 Child- and Adolescent Psychiatry, LUMC-Curium, Endegeesterstraatweg 27, Oegstgeest, The Netherlands

**Keywords:** Autism, Scalp hair cortisol, Stress, Mental health, Behavior problems, Eating behavior, BMI, Obesity

## Abstract

Children with autism and their parents face daily challenges that may be stressful for both. However, little is known about biological stress (hair cortisol concentrations [HCC]) in these families and its connection to children’s health outcomes. This study investigates biological stress in children with autism and their parents and its associations with child mental health, eating behavior and BMI. Stress was measured in 102 young children with autism and their parents (101 mothers, 86 fathers) using HCC and self-reported parenting stress (OBVL). Child mental health was measured through autism symptoms (ADOS-2, SRS-2) and problem behavior (CBCL). Child eating behavior (CEBQ) and BMIz were also measured. Children with autism had higher HCC than their peers. Child HCC was not linked to mental health, eating behavior, or BMIz. Maternal stress (self-reported and HCC) was associated with child problem behavior. In fathers, self-reported parenting stress correlated with child autism symptoms (SRS-2) and behavior problems. Both parents’ self-reported stress was associated with child eating behavior, specifically emotional undereating and overeating. In conclusion, higher HCC levels in children with autism in comparison to children from the general population, suggest differences in stress-regulation in children with autism. Given these findings, monitoring HCC in research and clinical practice could improve our understanding of stress-regulation in children with autism. The association between parental stress and children’s mental health and eating behaviors, underscores the importance of considering family dynamics in clinical (preventive) interventions and in further research that addresses the mental and physical health of children with autism.

**Supplementary Information:**

The online version contains supplementary material available at 10.1007/s10803-024-06672-0.

Children with Autism Spectrum Disorder (ASD) face everyday challenges that may impact family life, which may be stressful for both children and their parents. Extensive research demonstrates that stress can have a negative impact on health, by triggering autonomic and hormonal responses and by influencing health-related behavior (O’Connor et al., 2021). Moreover, parental stress may elevate these effects, as it can negatively affect the child’s mental and physical health. A meta-analysis demonstrated the association between parental stress and children’s emotional and behavioral problems in school-aged children (Ribas et al., 2024). Recent studies show that children with autism and adults have higher rates of physical health problems than children from the general population, such as obesity, gastrointestinal problems, and worse perceived metabolic health (Sammels et al., 2022; Warreman et al., 2023). In addition, comorbid mental health problems are common in individuals with autism. To illustrate: about 70% of the children with autism have at least one comorbid disorder (Simonoff et al., 2008). Studies also demonstrate higher mortality risks in individuals with autism (Catalá-López et al., 2022). As stress may contribute to both mental- and physical health problems, it is important to further investigate how these are related to each other. However, research regarding the associations between stress of children with autism and their parents and the health of individuals with autism, specifically during early childhood is sparse. This is particularly important as the patterns that are formed during early childhood can have lasting effects throughout the individual’s life. Therefore, the current study will investigate biological stress in children with autism and their parents and explore its relationship to the mental health, eating behavior and BMI in children with autism.

The hypothalamic-pituitary-adrenal (HPA) axis is activated during stressful situations and forms a chain reaction, which results in the release of the glucocorticoid cortisol. The HPA-axis is in important mechanism for coping with challenges. However, if challenges become overwhelming or chronic, this can lead to a dysregulation of the HPA-axis. Previous studies associate higher HPA-axis activity with various indices of chronic stress and physical health problems, including obesity (Dettenborn et al., 2010). When studying biological stress, serum and salivary cortisol are the most common measures. However, these measures only reflect short-term HPA-axis activity. Given that individuals with autism and their parents face ongoing daily challenges, investigating long-term HPA-axis activity may increase our understanding of the biological stress in children with autism and their parents.

Studies that have compared HCC of children with autism to same-aged children from the general population have produced mixed findings (Lin et al., 2024; Ogawa et al., 2017). The study of Ogawa and colleagues (2017) found higher HCC levels in children with autism than same-aged typically developing children, but this study was performed in a small sample of 28 children with autism. Lin and colleagues conducted a larger study that did not find differences between children with autism (*n* = 307) and without autism (*n* = 282). However, this study was performed in a broad age-range (2 to 17 years). Investigating stress in young children specifically may be valuable, as this may provide insights on how stress may impact the development of children’s health at an early age. Currently, no studies have specifically investigated HCC in young children with autism.

Previous studies in the general population show a link between stress exposure in early childhood and mental and physical health problems during adolescence, such as depression and obesity (Danese & Tan, 2014; Hazel et al., 2008). Stress could be connected to children’s health through biological mechanisms. For example, previous studies found associations between HPA-axis functioning and overweight in school-aged children and adolescents (Miller & Lumeng, 2018). Another mechanism through which stress could be related to the health of children with autism, is through behavioral pathways. Children may use eating behavior to cope with stress, resulting in unhealthy eating habits. In our previous study, we found a positive association between child BMIz and food approach behavior in the child (van der Lubbe et al., 2024). Notably, we found 8 times higher obesity rates in children with autism compared to the general population (van der Lubbe et al., 2024). In addition, previous studies demonstrate that individuals who have experienced high levels of stress in early childhood are at risk for later behavioral problems (Rudd et al., 2021). As research indicates that early life stress can have an impact on the health of individuals, it is important to explore possible associations between stress and children’s health early in life.

Another factor that may be associated with child health, is the stress of their parents. For example, a previous study found a link between reported parenting stress in mothers and internalizing and externalizing problem behavior of their children with autism (Zaidman-Zait et al., 2014). Furthermore, stress of parents may be associated with the health of their children in various ways. First, health problems of children may increase stress of their parents. On the other hand, parenting stress may also increase health problems in their children. In a previous study, we found an association between self-reported parenting stress and disinhibited eating behavior of mothers of young children with autism (van der Lubbe et al., 2022). Additionally, another study demonstrated a negative relationship between parental stress and healthy food availability at home, which may impact healthy eating behavior of their children (Jang et al., 2021). While most research on this subject has focused on subjective reports of parental stress, fewer studies have examined the incidence of biological stress. A previous study in children susceptible to obesity, reported an association between high maternal scalp hair cortisol concentrations (HCC) and high fat mass as well as low fat free mass in their children (Larsen et al., 2016). However, less is known about the associations between stress of parents and the mental health, eating behavior and BMI of their children with autism. Furthermore, most research has focused on mothers, while there may be associations of child health with stress in fathers as well. A better understanding of these associations is specifically important, as mothers and fathers of young children with autism report high rates of clinical parenting stress compared to parents of neurotypical children (van der Lubbe et al., 2022).

The current study investigates stress of young children with autism and will explore associations between stress of children with autism with stress of their parents and child mental health, eating behavior and BMI. The first goal of this study is to investigate whether biological stress levels of children with autism differ from same-aged children from the general population. The second goal of this study is to explore associations between biological stress of children with autism and their mental health, eating behavior and BMI. The third goal of the study is to explore associations between stress (self-reported and biological) of parents of children with autism and their children’s mental health, eating behavior and BMI.

## Method

### Procedure

The current study is a cross-sectional study investigating stress in young children with autism and their parents. This study is part of the ongoing Tandem Study (Dutch Trial register: NL7534), approved by the Institutional Review Board of the Leiden University Medical Center, The Netherlands. Data were collected between 2018 and 2024.

### Participants

As the current study is part of an ongoing randomized controlled trial (RCT), the participants in the current study overlap with those reported in our previous study (van der Lubbe et al., in press). Families were recruited from Youz Parnassia Group, GGZ Delfland and Jonx, all Dutch mental health care providers. Families were eligible for inclusion if: (1) the child was diagnosed with ASD, (2) the child was aged between 3 and 7 years and (3) parents could understand Dutch without the help of a translator. Children who started new psychotropic medication three months prior to participating in the study were excluded.

## Measures

### Stress

#### Hair Cortisol Concentrations (HCC)

Hair samples of approximately 100 hairs were cut from the posterior vertex of the scalp, as close to the scalp as possible in children and their parents. The most proximal 3 cm of the hair strands were used, which corresponds to a period of three months. After collection, hair samples were stored at room temperature and sent to the Erasmus Medical Centre (EMC) for laboratory analysis. At the EMC, the hair samples were weighed, washed and cortisol was extracted with methanol. Next, hair cortisol was analysed using liquid chromatography-tandem mass spectrometry (LC-MS/MS). Parents were asked to complete a questionnaire regarding hair washing frequency, usage of hair products and the use of glucocorticoids in themselves and their children.

The reference intervals that were provided by the study of Kruijf and colleagues (2020) for children aged 3 to 7 years (*n* = 82) were used to determine cut-off scores for high and low HCC and used to compare our sample with the norms of de Kruijf and colleagues. This study focuses on an age-selected subgroup drawn from a general population sample of children aged 0 to 18 years (*n* = 625), who were recruited through infant-welfare centres and schools. We will refer to this subgroup as ‘peers’.

#### Reported Parenting Stress

Reported parenting stress was measured in mothers and fathers using the Parenting Stress Questionnaire (OBVL). The OBVL is a 34-item self-report measure of parenting stress (Vermulst et al., 2015). Mothers and fathers scored items on a 4-point Likert scale. For this study, the total score on the OBVL was used (*Cronbach’s alpha* = 0.91), in which a high score reflects a high level of parenting stress.

### Child Mental Health

#### Autism Symptoms

Autism severity was measured using the Autism Diagnostic Observation Scale (ADOS-2; de Bildt et al., 2008). The ADOS-2 is a standardized, semi-structured observational measure of ASD symptoms. For this study, we used the standardized ADOS severity score, ranging from 0 (minimal) to 10 (high), representing the severity of autism symptoms. The ADOS-2 consists of 4 modules, administered according to the level of expressive language of individuals. Modules 1, 2 and 3 were used for the current study.

Social Ability of the child was measured using the Social Responsiveness Scale – second edition (SRS-2; Constantino & Gruber, 2012). The SRS-2 is a 65-item questionnaire consisting of 5 subscales (Social Awareness, Social Cognition, Social Communication, Social Motivation, Restricted Interests and Repetitive Behavior) and a total-score measuring the severity of social deficits in children with autism. The SRS-2 was completed by both parents. The Dutch version of the parent report SRS-2 demonstrated high internal consistency (Cronbach’s alpha = 0.92–0.95).

#### Child Problem Behavior

Child problem behavior was measured using the Child Behavior Checklist (CBCL; Achenbach & Rescorla, 2000; Achenbach & Rescorla, 2001). The CBCL is a caregiver report form targeting problem behavior in children, using two versions: the preschool version (CBCL/1½-5), containing 100 problem behavior questions and the school-age version (CBCL/6–18), containing 118 problem behavior questions. Parents rated their child’s problem behavior on a 3-point scale, with higher scores reflecting a higher level of the corresponding behavior. For the current study, the total raw score and the raw scores on the subscales Internalizing and Externalizing problems were used. As both versions have a different number of items, the total raw score on each subscale was divided by the number of items for comparability between the two versions. The CBCL demonstrates strong internal consistency (Cronbach’s alpha > 0.80) and has been validated for use with children with autism (Pandolfi et al., 2009, 2012).

### Child Eating Behavior and Body Mass Index

#### Child Eating Behavior

Child eating behavior was measured using the Child Eating Behavior Questionnaire (CEBQ). The CEBQ is a 35-item questionnaire consisting of 8 subscales measuring food approach behaviors (subscales: Food Responsiveness, Enjoyment of Food, Emotional Overeating and Desire to Drink) and food avoidant behaviors (subscales: Satiety Responsiveness, Slowness in Eating, Emotional Under-Eating and Food Fussiness). Mothers rated items on a 5-point Likert scale, with higher scores indicating a higher level of the specific behavior. The CEBQ has good psychometric properties in terms of factor structure, internal reliability and correlations between subscales (Sleddens et al., 2008). Cronbach’s alpha values for the subscales range from 0.75 to 0.91 (Wardle et al., 2001).

#### Body Mass Index

Body height was measured using a stadiometer (Seca 213), and body weight by a digital scale (Seca Clara 803) in all children. Body Mass Index (BMI) was calculated by dividing weight in kilograms by the square of height in meters. Child BMI was standardized to BMIz, using Growth Analyser Software Research Calculation Tools version 4.1.5 with the Fifth National Dutch Growth Study as a reference group. Based on international cut-off points by Cole and colleagues (2000), children were classified into three BMI classes: healthy weight, overweight and obese. The percentage of participants in each category was compared to Dutch children aged 2–21 years (*n* = 20.867) from the Fifth Dutch Growth Study, the actual standard of comparison in Dutch pediatric health care.

#### Demographic Variables

Parents indicated their highest completed education and their birth country. The highest completed education of mother was used as a measure of Social Economic Status (SES). Children were categorized into one of the two categories: (1) Non migration background and (2) Migration background (if one- or both parents was born outside the Netherlands).

### Statistical Analyses

To investigate whether children with autism show differences to their peers regarding biological stress, Chi-Square Goodness of Fit tests were performed. The reference intervals that were provided by the study of de Kruijf and colleagues (2020) were used to determine cut-off scores for high and low HCC and used to compare our sample with the norms of de Kruijf and colleagues. Additionally, we used the Mantel-Haenszel test to control for sex and age of the children.

For our next research question, we explored whether biological stress of the child was associated with child mental health, eating behavior and BMI. As child HCC was right-skewed, we performed a Spearman’s correlation analysis. We used maternal reports of the SRS, CBCL and CEBQ. If mothers did not complete questionnaires, we used the questionnaires of fathers.

For our last research question, we explored whether stress (self-reported and biological) of the parents was associated with child mental health, eating behavior and BMI. As HCC of mothers and fathers was right-skewed, we performed a Spearman’s correlation analysis. For associations between maternal HCC and the SRS, CBCL and CEBQ, we used maternal reports. We used paternal reports for correlations between HCC of fathers and SRS, CBCL and CEBQ.

For each analysis, we performed a sensitivity analysis by excluding the HCC of individuals who used corticosteroids. Missing values were treated using pairwise deletion. All analyses were performed in SPSS Statistics 27.

## Results

### Descriptives

One hundred two children with autism (85 boys, 17 girls) aged 3 to 7 years (Median = 5.1, IQR = 2.1) and their parents (101 mothers, 86 fathers) participated. Autism severity scores (ADOS) raged from 1 to 10 (Median = 6, IQR = 3). In our sample, 11.3% of the children was overweight and 13.4% of the children was obese. Additional details regarding weight-classes and sociodemographic characteristics of our sample, compared to the general population, are displayed in Table 1. Additionally, mean and median values of HCC and the mental health, eating behavior and BMI measures are displayed in Supplementary Table 1.

**Table 1:** Sociodemographic characteristics and weight-status of children with autism aged 3–7 years (*n* = 102) compared to the general population

	Children with autism		Comparison group			
	*N*	%	%	Chi-square	*p*	Reference group
Highest completed educational level of mother				3.85	0.15	Dutch females (*n* = 2,218,000) aged 25 to 45 years from the Dutch general population^b^
Low	11	12.2	10.9			
Middle	38	42.2	33.4			
High	41	45.6	55.6			
Ethnic background of the child^a^				8.21	< 0.001	The Dutch population (*n* = 17,591,000)^c^
Dutch	60	63.2	75.8			
Migration background	35	36.8	24.2			
Weight-status				61.17	< 0.001	Dutch children aged 2–21 years from Fifth National Growth Study^d^
Overweight	11	11.3	12.1			
Obesity	13	13.4	2			

^a^Children were classified with a migration background if one or more parents was born in a different country than the Netherlands; ^b^Centraal Bureau voor Statistiek, (2021); ^c^Centraal Bureau voor Statistiek, (2022); ^d^Schönbeck & van Buuren, (2010)

There were 11 children, 3 fathers and 11 mothers who used corticosteroids and there were 13 children and 26 fathers with hair strand shorter dan 3 centimeters. However, excluding these cases from analysis did not make a difference in results regarding the association between HCC and the other variables. Therefore, analyses were performed including these cases to have sufficient power for the study. Data was collected between November 2018 and April 2024.

### Biological Stress in Children with Autism Versus Peers

As shown in Tables 1 and 25.3% of the children with autism scored above the 97.5th percentile for scalp hair cortisol, while 2.5% of the children in the reference group scored above the 97.5th percentile (*χ*^2^ = 8.92, *p* <.01). The difference remained significant when children who used corticosteroids or with hair strands shorter than 3 cm were excluded from analysis. Differences between children with autism and the reference group remained significant if controlled for gender (*χ*^2^ = 4.88, *p* =.01) and age (*χ*^2^ = 7.34, *p* <.01) of the children by using the Mantel-Haenszel test. Frequencies and percentages of children with autism above the 97.5th percentile are summarized in Supplementary Table 2.

**Table 2:** Scalp hair cortisol in children with autism (3–7 years) compared to their peers (De Kruijf et al., 2020)

	Children with autism (*n* = 98)		Reference group (*n* = 82)		
	N	%	%	χ^2^	*p*
Normal range	83	84.7	97.5	8.92	< 0.01
>=97.5th age percentile	15	15.3	2.5		

### Associations Between Child Stress and Their Mental Health, Eating Behavior and BMI

As presented in Table 3, child HCC was not associated with autism symptoms and problem behaviour in young children with autism. Furthermore, as shown in Table 4, there was no significant correlation between child HCC, eating behaviour and BMIz.

**Table 3:** Spearman’s correlations between HCC of children with autism and autism severity and behavioral problems

	HCC Child 3–7 years of age	
*Autism Symptoms*	*r*	*p*
Autism Severity (ADOS-2*)*	0.03	NS
Social Awareness (SRS-2)	0.02	NS
Social Cognition (SRS-2)	0.01	NS
Social Communication (SRS-2)	0.10	NS
Social Motivation (SRS-2)	0.05	NS
Restricted Interests and Repetitive Behavior (SRS-2)	0.01	NS
Total autism symptoms (SRS-2)	0.02	NS
*Child problem behavior*		
Externalizing behavioral problems (CBCL)	− 0.05	NS
Internalizing behavioral problems (CBCL)	− 0.05	NS
Total behavioral problems (CBCL)	− 0.08	NS

Abbreviations: ADOS-2 = Autism Diagnostic Observation Scale-Second Edition; CBCL = Child Behavior Checklist; NS = Non-significant; HCC = Hair Cortisol Concentration; SRS-2 = Social Responsiveness Scale-2

**Table 4:** Spearman’s correlations between HCC of children with autism and eating behavior and body Mass Index

	HCC Child 3–7 years of age	
*Child eating behavior (CEBQ)*	*r*	*p*
Food responsiveness	− 0.07	NS
Emotional overeating	0.11	NS
Enjoyment of food	− 0.03	NS
Desire to drink	− 0.02	NS
Satiety responsiveness	− 0.07	NS
Slowness in eating	− 0.10	NS
Emotional undereating.	− 0.02	NS
Food fussiness	0.01	NS
*Physical Measurements*		
Body Mass Index - SD	0.05	NS

Abbreviations: BMIz = Standardized Body Mass Index; CEBQ = Child Eating Behavior Questionnaire; NS = Non-significant; HCC = Hair Cortisol Concentration

### Associations Between Parental Stress and Child Mental Health, Eating Behavior and BMI

As shown in Table 5, there was a negative association between maternal HCC and externalizing problem behavior (*r* = −.29, *p* <.01), internalizing problem behavior (*r* = −.22, *p* =.04) and total problem behavior (*r* = −.25, *p* =.02) of their children, while we found a positive correlation between reported parenting stress of the mothers and externalizing problem behavior (*r* =.37 *p* <.01), internalizing problem behavior (*r* =.29, *p* = < 0.01) and total problem behavior (*r* =.38, *p* <.01) of their children. Associations between maternal HCC and child mental health remained significant after excluding mothers who used corticosteroids from HCC analysis. Stress of mothers was not significantly associated with autism symptoms of their child.

**Table 5:** Spearman’s correlations between stress of parents and autism severity and behavioral problems of the child

	Mothers		Fathers	
	HCC	OBVL	HCC	OBVL
*Child Autism Symptoms*				
Autism Severity (ADOS-2*)*	0.00	− 0.06	− 0.16	− 0.15
Social Awareness (SRS-2)	0.00	0.19	0.08	0.29*
Social Cognition (SRS-2)	0.04	0.01	− 0.12	0.24*
Social Communication (SRS-2)	0.03	0.10	− 0.03	0.28*
Social Motivation (SRS-2)	− 0.01	0.22*	− 0.01	0.40***
Restricted Interests and Repetitive Behavior (SRS-2)	− 0.04	0.11	− 0.01	0.38***
Total autism symptoms (SRS-2)	− 0.01	0.16	− 0.03	0.36**
*Child problem behavior*				
Externalizing behavioral problems (CBCL)	− 0.29**	0.37***	− 0.11	0.47***
Internalizing behavioral problems (CBCL)	− 0.22*	0.29**	0.07	0.40***
Total behavioral problems (CBCL)	− 0.25*	0.38***	− 0.01	0.49***

Abbreviations: ADOS-2 = Autism Diagnostic Observation Scale-Second Edition; CBCL = Child Behavior Checklist; HCC = Hair Cortisol Concentration; OBVL = Parenting Stress Questionnaire; SRS-2 = Social Responsiveness Scale-2. *p <.05, **p <.01, ***p <.001

In fathers, we did not find a significant correlation between HCC and child problem behavior or autism symptoms. Reported parenting stress of the fathers was positively associated with reported autism symptoms of the child all domains of the SRS-2 (*r* =.24-0.40, *p* <.05). Furthermore, reported parenting stress of fathers correlated positively with externalizing problem behavior (*r* =.47, *p* <.01), internalizing problem behavior (*r* =.40, *p* <.01) and total problem behavior (*r* =.49, *p <*.01) of their children.

As displayed in Table 6, there was a positive correlation between reported parenting stress of mothers and both emotional overeating of the child (*r* =.22, *p* =.03) and emotional undereating of the child (*r* =.42, *p* <.01). Reported stress of mothers did not correlate with any of the other subscales of the CEBQ and child BMIz. Additionally, there was no correlation between maternal HCC and eating behavior or BMIz of the child.

**Table 6:** Spearman’s correlations between stress of parents and eating behavior and body Mass Index of the child

	Mothers		Fathers	
	HCC	OBVL	HCC	OBVL
*Child eating behavior (CEBQ)*				
Food responsiveness	− 0.09	0.12	0.00	0.25*
Emotional overeating	− 0.02	0.22*	− 0.17	0.27*
Enjoyment of food	0.13	− 0.06	0.05	0.08
Desire to drink	− 0.02	− 0.01	− 0.03	− 0.07
Satiety responsiveness	− 0.17	0.12	0.01	0.22*
Slowness in eating	− 0.10	0.17	0.13	0.22
Emotional undereating.	− 0.20	0.42***	− 0.19	0.35**
Food fussiness	− 0.15	0.11	− 0.03	0.05
*Physical Measurements*				
Body Mass Index – SD	− 0.07	− 0.00	0.09	0.05

Abbreviations: BMIz = Standardized Body Mass Index; CEBQ; Child Eating Behavior Questionnaire; HCC = Hair Cortisol Concentration; OBVL = Parenting Stress Questionnaire;. *p <.05, **p <.01

In fathers, there was a positive association between reported parenting stress and child eating behavior on the following subscales of the CEBQ: food responsiveness (*r* =.25, *p* =.02), emotional overeating (*r* =.27, *p* = 02), satiety responsiveness (*r* =.22, *p* =.05) and emotional undereating (*r* =.35, *p* <.01). Reported stress of fathers did not correlate with any of the other subscales of the CEBQ and child BMIz. Additionally, there was no correlation between HCC of fathers child eating behavior and BMIz.

## Discussion

The current study investigated stress in children with autism and their parents and explored associations between stress and child mental health, eating behavior and BMI. While children with autism had higher HCC than their peers, biological stress of children with autism was not associated with their mental health, eating behavior and BMI. Stress of mothers, as well as stress of fathers, was related to child mental health, eating behavior and BMI.

Compared to their peers, children in our sample exhibited higher HCC, with 15.3% of the children scoring above the 97.5th percentile. This finding is in line with the study of Ogawa and colleagues (2017), that reported higher HCC in children with autism compared to same-aged typically developing children. However, their sample size was smaller than ours (34 children with AUTISM versus 102 in our study) and the children were older than the children in our group (mean age = 11.9 years versus 5.1 years in our study). Another recent study did not find differences between HCC of children with and without autism (Lin et al., 2024). The age range of the study by Lin and colleagues was broader than ours, with children who were aged between 2 and 17 years old, compared to the 3 to 7 years old age range in our study. Furthermore, the autistic group was significantly older than the non-autistic group in the study by Lin and colleagues. This age difference could explain why they did not find differences between the two groups, although their results indicated higher HCC concentrations in younger children. The age-related differences in HCC could be explained by both biological changes as well as psychosocial factors, such as family stress and socio-economic differences (Perry et al., 2022). In addition, the studies of Ogawa and colleagues (2017) and Lin and colleagues (2024) used another, less sensitive, analysis method for cortisol analysis (ELISA method) than our study (LC-MS method), which may explain differences in findings. The current study is the first study to demonstrate a difference between young children with autism and their peers. Further research is needed to better understand biological stress of children with autism.

While we did observe higher HCC values in children with autism compared to their peers, we did not find any associations between HCC of children with autism and their mental health, eating behavior or BMI. These results suggest that while children with autism may demonstrate elevated biological stress levels, the relationship with mental health, eating behavior and BMI measures may be more complex and needs further investigation. Given that the children in our study were aged between 3 and 7 years old, a developmental stage where children are still very dependent on their parents, it could be hypothesized that parents, rather than children themselves, may have a large impact on the mental- and physical wellbeing of young children. As parental stress may affect parenting behavior and family environment, it could possibly play a more important role in shaping the child’s mental health and eating behavior than the child’s own stress levels. This is in line with a study by Perry and colleagues (2022) that found an association between HCC of mothers and that of their children, especially in young children. This hypothesis is supported by our previous study in the same sample, that found a significant correlation between HCC of parents and HCC of children with autism (van der Lubbe et al., in press). Therefore, longitudinal research is warranted to further investigate these multifactorial relationships.

We found an association between reported parenting stress in mothers and fathers and problem behavior of children with autism. This is in line with previous research, that demonstrates an association between parental stress and externalizing and internalizing behavioral problems of the child (Barraso et al., 2018). Hastings (2002) proposes a bidirectional model in which behavioral problems elevates parenting stress, which disrupts parenting behavior, further elevating the behavioral problems of the child. This bidirectional model is inconsistently supported by research, with some studies finding evidence for a bidirectional relationship between parenting stress and behavioral problems in children with autism, while others support a unidirectional model in which parental stress predict later behavioral problems of the children or vice versa (Yorke et al., 2018; Zaidman-Zait et al., 2014).

Interestingly, children with a higher level of behavioral problems had mothers with lower HCC. In other words, the biological stress response of mothers seems to be lower when their children demonstrate more behavioral problems. A possible explanation is that chronic stress leads to a dampening of the HPA-axis. This is in line with our previous study in the same sample, that demonstrated a negative correlation with reported parenting stress and HCC of parents (van der Lubbe et al., in press). Moreover, Radin and colleagues (2019) found lower HCC in mothers of children with autism compared to mothers of typically developing children. These findings demonstrate the complex interplay between maternal stress regulation and child behavioral problems, suggesting that behavioral problems in children may be associated with a dampened physiological stress response in mothers. However, as the current study was cross-sectional, it is relevant to further investigate these relationships longitudinally.

While self-reported parenting stress for both mothers and fathers correlated with mental health of their children, and maternal HCC was associated with mental health of their child, we did not find this correlation for HCC of fathers. This means that while behavioral problems and autism symptoms are associated with reported parenting stress in fathers, they are not necessarily associated with biological stress in fathers. Possibly, the psychological response to behavioral problems of the child may precede the biological response, therefore it may be that the connection develops later in time. Additionally, differences in exposure to childcare responsibilities among fathers could influence the extent to which the parenting stress translates to a biological stress response. Previous research demonstrated that fathers of children with autism spend approximately 26% less time in childcare than mothers (Rodriguez et al., 2019). Another explanation is that the biological response of the HPA-axis may differ between fathers and mothers. Previous studies in various species suggest that the physiological reactivity to stress is greater in females than in males (Goel et al., 2014). This could explain why we found a correlation between reported parenting stress and mental health problems of the child, but not between these factors and HCC of fathers.

Although almost 25% percent of the children in our study were overweight or obese, BMI was not related to children’s or parents’ stress levels. However, in both mothers and fathers, we found associations between reported parenting stress and eating behavior of the child. This is in line with previous research in children without autism, that found a correlation between parenting stress and child’s eating behavior (Jang et al., 2021). Interestingly, parenting stress was associated with emotional overeating and emotional undereating of the child. It is possible that both emotional overeating and emotional undereating leads to increased parenting stress, due to the health risks that are associated with both eating behaviors. On the other hand, it is also possible that both emotional overeating and emotional eating is a reaction of the children to the stress they experience due to the stress of their parents. Our previous study in the same sample indicated a correlation between HCC of parents and HCC of children with autism (van der Lubbe et al., in press). While children may react to stress by using food as a source of comfort, leading to overeating, children may also experience reduced appetite, leading to undereating. As stress may influence children’s behavior in diverse ways, this may explain why we did not observe a relationship between BMI and the stress levels of children with autism and their parents.

The current study has some limitations. One limitation is that our study design was cross-sectional and therefore, we cannot make conclusions about causality based on our results. Additionally, for future research it is important to acknowledge that the mental health, eating behavior and BMI of children with autism may be associated with other underlying factors too, such as genetic health conditions. For example, a previous twin-study in children with autism found that shared genetic factors may contribute to both ASD and emotional symptoms (Tick et al., 2015). However, as the mental health, eating behavior and BMI of young children with autism is a complex interplay of genetic, environmental and developmental factors, the current study may help by identifying specific factors that are associated with the mental health, eating behavior and BMI of children with autism. We consider it a strength of the study that we used an integrated approach, in which concurrently mental- and physical measures were used.

The current study investigated biological stress of children with autism and explored associations between stress of children with autism and their parents, as well as child mental health, eating behavior, and BMI. First, we found higher levels of HCC in children with autism compared to their peers. Second, we did not find associations between child HCC, mental health, eating behavior and BMI. Third, parenting stress was associated with behavioral problems of the child, autism symptoms and with child eating behavior. Differences in mothers and fathers between their associations of stress with child factors motivate the urge to better understand underlying mechanisms in further research. We encourage future studies to examine longitudinal effects of stress on mental health, eating behavior and BMI of children with autism. For example, it would be relevant to further investigate whether the amount of time attributed to parenting tasks, such as time spent caregiving or other parenting tasks, may impact parental stress or health. Additionally, it would be significant to investigate the effect of interventions on (parenting) stress and mental health, eating behavior and BMI of children with autism.

## Conclusion

In the current study, differences in HCC between young children with autism and their peers were found, suggesting potential alterations in stress-regulation of young children with autism. While the associations between biological stress and child mental health, eating behavior and BMI were not observed, we found a significant relationship between stress of parents (self-reported and biological) and child mental health and eating behavior.

These findings demonstrate the importance of addressing stress of parents in research and clinical care of children with autism, as this may have an impact on the mental health and eating behavior of their children. For example, interventions directed at reducing parenting stress could be beneficial for the mental and physical health of both parents and children. Additionally, it could be beneficial to monitor HCC in research and clinical practice, as this could improve our understanding of stress-regulation in children with autism

## Electronic supplementary material

Below is the link to the electronic supplementary material.

Supplementary Material 1
