# Supplementary material for: Hair Cortisol in Young Children with Autism and Their Parents: Associations with Child Mental Health, Eating Behavior and Weight Status
Source: J Autism Dev Disord. 2025 Jan 22;56(6):2354–63. doi: 10.1007/s10803-024-06672-0 (PMC13222298; doi:10.1007/s10803-024-06672-0)
Supplement: Supplementary file 1 — Supplementary Material 1 [file 10803_2024_6672_MOESM1_ESM.docx]

| Table S1. Descriptive values of hair cortisol concentrations and mental health, eating behavior and BMI of young children with autism. | | |
| --- | --- | --- |
| **Measure** | **n** | **M (SD)** |
| Hair cortisol concentration (pg/mg)^a^ | 95 | 2.1 (6.03) |
| Autism Severiy Score (ADOS-2) ^a^ | 97 | 6.0 (3.00) |
| Total score SRS-2 | 90 | 93.8 (2.58) |
| Total behavioral problems (CBCL) | 94 | 67.4 (9.01) |
| Food Responsiveness (CEBQ)^a^ | 92 | 11.0 (8.00) |
| Emotional Overeating (CEBQ)^a^ | 92 | 6.0 (4.75) |
| Enjoyment of Food (CEBQ)^a^ | 92 | 13.0 (5.00) |
| Desire to Drink (CEBQ)^a^ | 94 | 7.0 (4.00) |
| Emotional Undereating (CEBQ)^a^ | 92 | 11.0 (8.00) |
| Satiety Responsiveness (CEBQ) | 91 | 14.2 (4.11) |
| Slowness in Eating (CEBQ)^a^ | 91 | 12.0 (7.00) |
| Food Fussiness (CEBQ)^a^ | 92 | 22.0 (9.00) |
| BMIz | 97 | 0.9 (1.52) |
| ^a^Variable was non-normally distributed, median and IQR’s are displayed.  Abbreviations: ADOS-2 = Autism Diagnostic Observation Scale-Second Edition; BMIz = Standardized Body Mass Index; CBCL = Child Behavior Checklist; CEBQ; Child Eating Behavior Questionnaire; HCC = Hair Cortisol Concentration; M = Mean; SD = Standard Deviation; SRS-2 = Social Responsiveness Scale-2. | | |

| Table S2. Frequencies of children above the 97.5^th^ percentile for hair cortisol categorized by sex and age-group. | | |
| --- | --- | --- |
|  | Above the 97.5^th^ percentile (%) | Within the normal range |
| Boys | 14 (17.1) | 68 (82.9) |
| Girls | 1 (6.3) | 15 (93.8) |
| 3-year-olds | 4 (16.7) | 20 (83.3) |
| 4-year-olds | 3 (12.0) | 22 (88.0) |
| 5-year-olds | 3 (15.0) | 17 (85.0) |
| 6-year-olds | 5 (17.9) | 23 (82.1) |
| 7-year-olds | 0 (0.0) | 1 (100.0) |
